# Evaluating the effect of an e-learning program designed to teach health care practitioners to inform patients with a model drawn from motivational interviewing: A pilot randomized controlled trial

**DOI:** 10.1016/j.pecinn.2026.100487

**Published:** 2026-07-08

**Authors:** Cristiana Fortini, Nina Rodriguez, Joseph Studer, Nicolas Bertholet, Jean-Bernard Daeppen, Jacques Gaume

**Affiliations:** a Addiction Medicine, Department of Psychiatry, Lausanne University Hospital and University of Lausanne, Lausanne, Switzerland; b Service of Adult Psychiatry North-West, Department of Psychiatry, Lausanne University Hospital and University of Lausanne, Switzerland

**Keywords:** Patient information, Communication skills training, Provider-patient communication, Motivational interviewing, E-learning, Pilot randomized controlled trial

## Abstract

**Objectives:**

We tested the effect of a 1.5-h e-learning course, which presents a new model for information-giving based on Motivational Interviewing, on practitioners' skills to inform patients.

**Methods:**

Thirty-two physicians and nurses at Lausanne University Hospital participated in a pilot randomized controlled trial to assess the impact of the e-learning on performance during an encounter with a simulated patient (SP). Participants were randomized (1:1) to complete the e-learning before (intervention) or after (control) the SP encounter. Encounters were coded using an adapted version of the validated MITI (Motivational Interviewing Treatment Integrity) coding scale. Analyses compared group differences and explored effects by clinical experience.

**Results:**

The intervention group showed significantly better communication behaviors when delivering information, using less persuasion and more collaborative strategies (e.g., asking permission, checking understanding, eliciting patient reaction). Effects varied by experience level, with less experienced practitioners showing greater effects in empathy, partnership, seeking collaboration and use of open questions.

**Conclusion:**

The e-learning impacted the way providers inform patients and shows promise as an effective tool for skill development, especially among less experienced practitioners.

Innovation: This study introduces a novel framework for conceptualizing and teaching information delivery as a structured interactive process and provides an institution-wide program for all staff involved in patient communication.

## Highlights

• Practitioners could benefit from clearer guidance when informing patients.
• An e-learning for information-giving based on Motivational Interviewing was tested.
• This pilot trial indicated that the e-learning significantly impacted tested skills.
• The intervention group showed less persuasion and more collaborative strategies.
• Improvements were observed especially among less experienced practitioners.

## 1 Introduction

Providing information is an essential element of what is considered today good provider-patient communication and is pivotal in the delivery of patient care [1], [2], [3]. However, effective transfer of information from practitioner to patient is challenging. It is a frequent cause of patient dissatisfaction and complaints [4], [5], [6], [7], and skills for engaging in such patient-centered communication vary among practitioners [8].

In a qualitative, exploratory study, we interviewed hospital health care professionals on how they perceive and experience delivering information to patients [9]. Practitioners reported taking a series of precautions before providing information, such as engaging the patient, creating a trusting relationship, gathering facts, eliciting prior knowledge etc. Yet they also expressed feelings of discomfort because they realized that delivering information is not as efficient as they expected and were eager for further training. We identified some possible confusions in the information delivery process. First, practitioners inform patients with the intention and the expectation that they adhere to and act on the information provided. Second, while they aim at patient action, they mostly use strategies that involve checking if the patient has understood the information, implying that understanding will ensure action.

Similar assumptions were also observed in a paper that examined commonly accepted practices of information transmission in health settings [3]. The authors showed, however, that people who receive health-related information do not unproblematically accept it and act upon it. Instead, they found respondents to question, challenge and resist information transfer, preferring instead to exercise considerable power and agency in how information was understood, integrated into their reality, and acted upon (or not). Furthermore, while studies show that understanding does not guarantee action [10] [11] [12], the quality of information provision is generally measured by the degree to which patients recall and understand the information [2] [13].

Yet, acting upon the provided information requires that the patient not only understand, but also accept what the practitioner suggests [14]. This requires the patient to be motivated to adhere, a state that is known to be influenced by the way the practitioner interacts with the patient [15]. In the context of delivering information, expecting patient action can push practitioners to use persuasive language, which can impinge on patients' sense of autonomy and elicit their defensiveness [16], [17]. This can lower motivation and intention to follow the aim of the message. Conversely, giving information with permission and supporting patient autonomy and exploration can decrease defensiveness and facilitate patient motivation and adherence [18], [19]. Motivational Interviewing (MI), a collaborative, evidence-based, person-centered approach that aims to strengthen motivation to change [15], encourages the use of an Ask-Offer-Ask approach to deliver information [20].

The Ask-Offer-Ask model resembles the Ask-Tell-Ask technique, a structured, patient-centered communication approach increasingly used in healthcare to facilitate information exchange by supporting autonomy, comprehension, and engagement [21]. The Ask-Tell-Ask involves asking for the patient's knowledge and needs, providing them with tailored information, and checking if the person has understood or has any questions [21], [22], [23], [24]. The MI Ask-Offer-Ask model, because it is embedded in an MI conversation, assumes that the provider adopts a specific posture that is in line with the MI spirit of partnership (letting go of an expert role), acceptance (respecting the person without judgement), compassion (giving priority to the well-being of the patient) and empowerment (adopting a strength-focused premise and supporting patient autonomy) [15]. This implies the use of specific strategies: a collaborative posture that ensures that the patient is ready and willing to receive the information; explicitly non persuasive and autonomy-supporting language when providing the information; and open questions that invite the patient to react to the information instead of just checking if they have understood it [20]. This last part is the most productive moment of the interaction, in that it allows the patient to explore their thinking around the information they received and to reinforce their motivation on what they intend to do with it. However, this is generally the part of the conversation that gets omitted [9], [25].

Both the results of our qualitative study and the literature led us to suggest that practitioners could benefit from clearer guidance in order to decrease discomfort and become more proficient at delivering information.

**Fig. 1:**
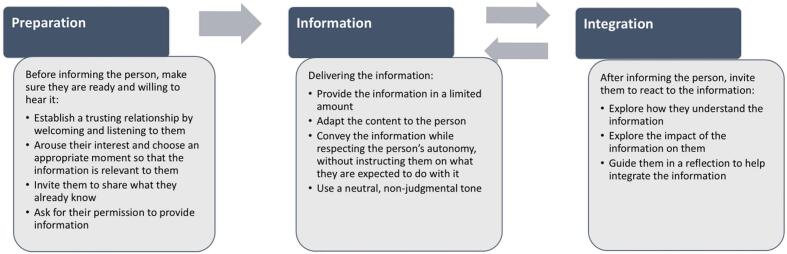
Information-delivery model, adapted from [15].

We developed an e-learning training program that presents a model for informing patients to support health care practitioners in this task. Because the focus was specifically on information-giving and therefore outside the context of MI, we adapted the Ask–Offer–Ask model and renamed its components to enhance clarity. This was considered essential to make the model accessible to all providers, including those without specific training in MI. The resulting model (Fig. 1) is described below.

• The first step is named “Preparation” to emphasize not only the importance of establishing a trusting relationship and building on the person's existing knowledge, but also of ensuring that the person is ready and willing to receive information before any is delivered.
• In the second step, named “Information”, we clarify the concept of supporting the person's autonomy by specifying that information should be delivered in a neutral tone, without instructions on what the patient is expected to do with the information.
• The third step is named “Integration” to emphasize and refine its focus. By explicitly highlighting the integration of information—how it is understood and what it implies for the patient—rather than simply checking the understanding of the information, the model offers practitioners clearer guidance on how to proceed after conveying information.

From this point onward, we are referring to this model as the “Preparation-Information-Integration” model. We also make explicit the possible transitions between the information and integration steps, highlighting the value of delivering one piece of information at a time and ensuring its integration before moving on to another. Two arrows—one going from information to integration and the other from integration to information —illustrate the potential back-and-forth movement between these two steps (Fig. 1).

The objective of this study is to test the effect of the e-learning on practitioners' skills to inform patients, with a pilot randomized design that assesses the performance of practitioners during an encounter with a simulated patient, using a psycholinguistic coding instrument to measure the skill of providing information according to the suggested model.

We hypothesized that practitioners who complete the e-learning would adopt the Preparation-Information-Integration model, therefore displaying higher measures of partnership (*foster power sharing rather than assuming the expert role), more use of collaboration-seeking behaviors* (e.g. ask permission, negotiate the agenda, elicit patient reactions), more use of non-persuasive and autonomy-supporting language (e.g. give information in a neutral manner, without instructions on what the patient is expected to do with it), more use of use open-ended questions (e.g. ask for patient's knowledge, opinions and reactions), less persuasive behavior and language. Finally, we investigated whether there were potential sub-group effects by stratifying regression analyses by clinical experience. This analysis was exploratory and had no directional hypotheses.

## 2 Methods

### 2.1 Setting

We conducted a pilot randomized controlled trial at the Lausanne University Hospital, Lausanne, Switzerland, a 1′500-bed institution which serves at the primary care center for the city of Lausanne and surrounding districts, and as a secondary and tertiary care center for the region and neighboring states.

The research protocol was approved by the institution's evaluation committee for research proposals (*CEDE - Commission d'évaluation des demandes d'enquêtes, in French*), authorization number CEDE 130.2024.

### 2.2 Participants

All registered nurses and physicians employed at CHUV were eligible for study inclusion. The project was presented to the hospital's medical and nursing directors and approved. They in turn communicated details of the study (either orally and/or with one or two rounds of emails) to the hospital departments' medical and nursing managers, encouraging them to forward the information to their teams and invite them to contact the first author if interested in taking part in the study. Participants were recruited on a voluntary basis. Given the pilot nature of the study, no data was gathered regarding the exact number of nurses and physicians that were notified of the study; this was dependent on each manager's willingness to pass down the information to their respective staff.

All interested participants provided online written consent for participation and completed a short online survey assessing socio-demographic variables and clinical background (see measures below). They were then randomly allocated (1:1) to the intervention or the control group through a computerized algorithm, stratified by profession to guarantee equivalent number of nurses and physicians in both study conditions. Participants in the intervention group *completed the e-learning first* and then, over the following week, met with a simulated patient for a 15-min interview (see details on interviews below). Participants in the control group conducted the same *interview with the simulated patient first* and then completed the e-learning in the following week.

### 2.3 *E*-learning training program

**Fig. 2:**
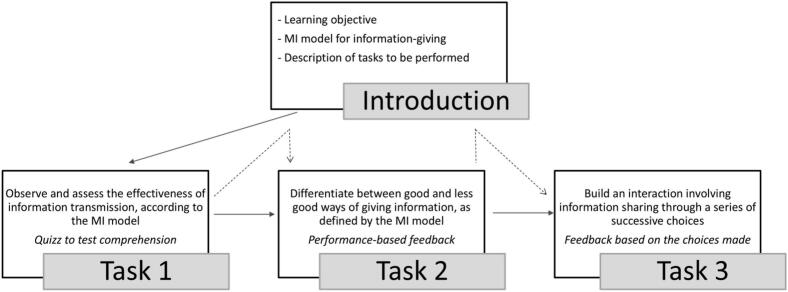
e-learning program.

The e-learning is built around a series of short video clips, each illustrating a practitioner informing a patient. The learner has 3 tasks to accomplish (Fig. 2): 1) observe an encounter in order to discover the model; 2) recognize the application of the model in various examples of conversations; 3) apply the model to construct an interaction through a series of options. Feedback is provided at each step. The training lasts approximately 1.5 h.

Participants completed the online training in one of the hospital's computer rooms. They were given instructions to access the e-learning online platform then completed it each at their own pace.

### 2.4 Meeting with a simulated patient

In the week following the training (intervention group) or preceding the training (control group), participants were invited to meet with a simulated patient. To ensure that the focus of the study remained on *how* participants delivered information rather than *what* they communicated, we selected a scenario involving sleep hygiene. This topic was intentionally chosen for its low medical complexity, broad applicability across healthcare professions, and lack of emotional or ethical sensitivity. Sleep hygiene is a common and relatable issue, familiar to both nurses and physicians, thereby minimizing performance differences due to clinical expertise or professional background [26], [27].

Interviews were held online using Webex videoconference software and were recorded in the software. One week before the interview, participants received written instructions (see Appendix A) indicating that they would conduct a 15-min interview with a simulated patient, a 46-year-old woman who, at the end of a routine consultation, raises concerns about sleep difficulties. The participants' task was to provide her with information about sleep hygiene. Participants were told that the focus of the study was on the way they would deliver the information rather than on the content of the information itself. Additional instructions included information about sleep hygiene and instructions on how to connect to the online platform.

The simulated patient was the same person for all interviews. She is a trained patient partner in therapeutic education and has over 6 years' experience as simulated patient in MI trainings. She was trained and supervised in her role by the first author (see Appendix B for role description).

### 2.5 Coding of interviews

The audio file of each recorded interview was extracted and transcribed using CORV, a software developed by the Lausanne University's Scientific Computing and Research Support Unit, based on automatic speech recognition algorithms, and optimized for French verbatim transcription. The interviews were coded on the transcripts while synchronously listening to the audio recordings.

A social sciences master-level student was trained to code the interviews. The coder then independently coded the recorded encounters. Coding challenges were addressed weekly in trainer-coder meetings, which lasted throughout the entire coding period.

A random sample of 10 sessions (about 30%) was double coded (rater + trainer) to assess inter-rater reliability. The coder was blinded to whether the sessions were simple- or double-coded. We used intraclass correlations (ICC) to measure inter-rater reliability, specifying a two-way random-effects model, absolute agreement, and individual measurement. All measures retained in the analyses had ICC above 0.75, indicating excellent inter-rater reliability [28].

### 2.6 Measures

At study entry, participants completed a short online survey assessing age (in years), self-identified gender (male, female, non-binary, refuse to answer), profession (nurse or physician), and clinical experience (defined as the number of years of practice in a clinical setting, i.e., involving contact with patients). There were also two questions related to knowledge about MI and therapeutic education. For both questions, there was a filler question asking whether they had any knowledge about the respective approach, and, if so, they were asked a multiple-choice question regarding how they acquired this knowledge using 4 categories: readings, awareness through courses/conferences, short specific training (<1 day), and specific training (>1 day).

Interviews were coded using an adapted version of the Motivational Interviewing Treatment Integrity (MITI) scale, version 4.2, which is a reliable and valid measure of Motivational Interviewing (MI) skills and is used as a fidelity measure in clinical trials [29], [30]. The MITI consists of global ratings and individual behavior counts. The global ratings are given based on an overall impression of the interaction, each scored on a 5-point Likert scale from 1 “low” to 5 “high”. Behavior counts are intended to capture specific behaviors without regard to how they fit into the overall impression of the practitioner's use of MI. Practitioner's speech is divided into utterances (complete thought or thought unit) and each utterance may receive only one behavior code.

A description of each MITI code is provided in Appendix C (second column). As the focus of the present study was on the task of providing information, we adapted the coding system for the purpose of this study. Details on these adaptations are provided in Appendix C (third column).

To test our hypotheses, we retained the following measures: the Partnership and Empathy global ratings (hypothesized to be higher in the intervention group), the behavior counts of Seeking collaboration, Seeking collaboration by eliciting exploration, Open questions, Giving neutral information, and Persuade with permission (hypothesized to be higher in the intervention group), the behavior counts of Persuade (hypothesized to be lower in the intervention group), and the summary scores of Percent open question, Ratio collaboration/information, Ratio exploration/information (hypothesized to be higher in the intervention group), and Percent persuade (hypothesized to be lower in the intervention group).

### 2.7 Data analysis

We first conducted standard descriptive analysis to describe our sample. We used Pearson's Chi^2^ test and Student's *t*-test to investigate whether there were significant differences between study groups on socio-demographic variables and clinical background.

To test our hypothesis that practitioners in the intervention group would adopt the Preparation-Information-Integration model when informing the simulated patient, we first compared study groups on the relevant MITI variables using box plots and Wilcoxon's rank-sum tests. We chose a non-parametric test and present median and 25th/75th percentiles since MITI measures were not normally distributed. We repeated this analysis using regression models adjusting for clinical experience. We used linear regression models with nonparametric bootstrap estimation specifying 1000 bootstrap replications to allow for non-normal variable distributions. We had planned to adjust for age and gender, but age was highly correlated with clinical experience (*r* = 0.96, *p* < 0.0001) resulting in multicollinearity, and gender was not equally distributed across groups (*n* = 1 male in intervention and *n* = 4 in control) resulting in frequent model failures. We did not adjust for profession, as randomization was stratified on this variable.

Finally, we investigated whether there were potential sub-group effects by stratifying regression analyses by clinical experience. Clinical experience was divided into 3 groups, based on distribution tertiles. Analyses stratified by clinical experience were adjusted for profession as profession was not necessarily equivalent within clinical experience sub-groups. We used bootstrap analyses as in non-stratified analyses. For all analyses, we considered significance level at *p* = 0.05.

## 3 Results

### 3.1 Sample

**Fig. 3:**
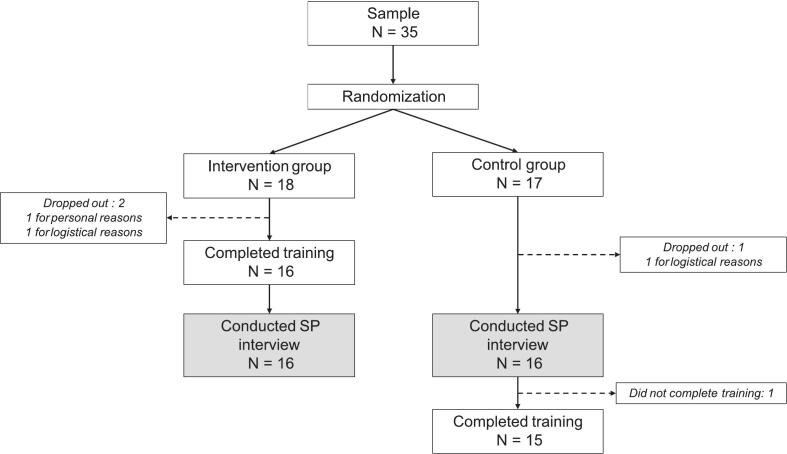
Flow chart of participant inclusion.

35 participants were included in the study and randomized into the intervention (*n* = 18) or control (*n* = 17) group (Fig. 3). After randomization, but before the assigned activities, two participants dropped out of the intervention group and one dropped out of the control group, for logistical or personal reasons, leaving 16 participants in each group. In the intervention group, 16 completed the e-learning then conducted the interview with the simulated patient (SP). In the control group, 16 participants conducted the SP interview, and 15 completed the e-learning after the interview.

Analyzed data therefore comprised 32 participants, 84% of which self-identified as female and 16% as male. Average age was 40.3 years old (standard deviation [SD] = 9.7). Half of the sample were nurses and half were physicians; 9 physicians were in the control group and 7 were in the intervention group. Participants had 14.5 years of clinical experience on average (SD = 9.3). About 56% had some knowledge of MI as well as of therapeutic education, mostly through lessons and conferences. A small number were formally trained in each approach. There were no significant differences between study groups on socio-demographic variables and clinical background (Table 1).

**Table 1:** Socio-demographic and clinical background, overall and by study condition.

	Control group (*N* = 16)		Intervention group (N = 16)		Total (*N* = 32)		Chi^2^/T a	p
Gender, N/% female	12	75.0	15	93.8	27	84.4	2.13	0.14
Profession, N/% physicians	9	56.3	7	43.8	16	50.0	0.50	0.48
Age, mean/SD	39.0	9.5	41.7	10.1	40.3	9.7	−0.78	0.44
Clinical experience, mean/SD	14.2	9.1	14.8	9.8	14.5	9.3	−0.19	0.85
Any knowledge in Motivational interviewing, N/%	10	62.5	8	50.0	18	56.3	0.51	0.48
-readings, N/%	2	12.5	1	6.3	3	9.4	0.37	0.54
-awareness (e.g. through courses, conferences), N/%	7	43.8	7	43.8	14	43.8	0.00	1.00
-short specific training (<1 day), N/%	1	6.3	1	6.3	2	6.3	0.00	1.00
-specific training (>1 day), N/%	2	12.5	1	6.3	3	9.4	0.37	0.54
Any knowledge in Therapeutic education, N/%	8	50.0	10	62.5	18	56.3	0.51	0.48
-readings, N/%	2	12.5	2	12.5	4	12.5	0.00	1.00
-awareness (e.g. through courses, conferences), N/%	6	37.5	6	37.5	12	37.5	0.00	1.00
-short specific training (<1 day), N/%	1	6.3	2	12.5	3	9.4	0.37	0.54
-specific training (>1 day), N/%	1	6.3	2	12.5	3	9.4	0.37	0.54

*Note*. SD: standard deviation.

a Chi^2^ test for categorical variables and t-test for continuous variables.

### 3.2 Comparison of participants' skills to inform patients across study groups

**Fig. 4:**
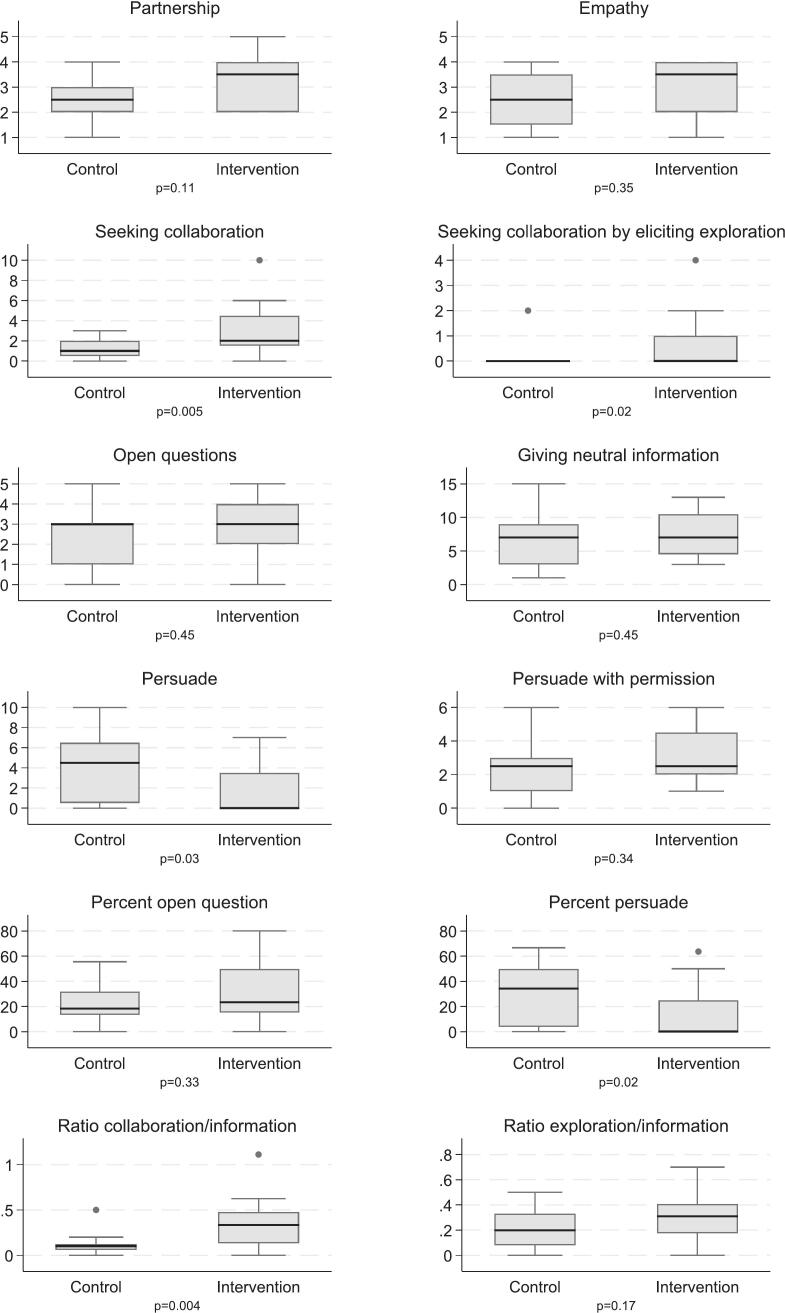
Comparison of the intervention and control groups on practitioners' skills to inform patients *Notes*: Box plot showing median (thick line), 25th to 75th percentile range (grey-shaded box), lower and upper adjacent values (whisker lines) and outside values (grey dots). *P* values based on Wilcoxon rank-sum test.

Fig. 4 depicts the comparison of the intervention and control groups on participants' skills to inform patients. No significant differences were found between intervention and control groups on the global ratings of Partnership and Empathy. Behavior counts indicated that participants in the intervention group used more skills that are recommended by the Preparation-Information-Integration model (Seeking collaboration, Seeking collaboration by eliciting exploration) and less of those unrecommended (Persuade). There were no significant differences for Open questions, Giving neutral information, and Persuade with permission. Regarding calculated summary scores, there were significant differences for Percent persuade and Ratio collaboration/information, indicating that participants in the intervention group used less persuasion and asked more for collaboration when they provided information. However, the Percent of open question and Ratio exploration/information did not differ significantly across groups.

All significant results were confirmed when replicating the analyses using regression analyses adjusting for clinical experience (Table 2). In addition, there was a significant effect for the global rating of Partnership, indicating higher level on this scale among the intervention group.

**Table 2:** Intervention effect on practitioners' skills to inform patients, adjusting for clinical experience.

Outcome variable	Coef.	BSE	p
Partnership	0.71	0.35	0.045
Empathy	0.39	0.44	0.37
Seeking collaboration	2.08	0.68	0.002
Seeking collaboration by eliciting exploration	0.58	0.28	0.04
Open questions	0.37	0.54	0.49
Giving neutral information	0.99	1.28	0.44
Persuade	−2.36	1.07	0.03
Persuade with permission	0.71	0.63	0.26
Percent open question	7.09	6.57	0.28
Percent persuade	−18.54	7.81	0.02
Ratio collaboration/information	0.23	0.08	0.002
Ratio exploration/information	0.10	0.06	0.12

*Notes*: Each line is a regression model, adjusting for clinical experience and using 1000 bootstrap replications. Coef: regression coefficient, BSE: bootstrapped standard error.

### 3.3 Intervention effect stratified by clinical experience

Despite the small sizes of the stratified samples, there were numerous significant differences among subgroups (Table 3). Results stratified by clinical experience showed significant effects in the expected direction on both global ratings of Empathy and Partnership among participants with low experience (0–9 years), while these effects were non-significant among participants with higher experience. A similar pattern was observed for Seeking collaboration, Open questions, Percent open questions, and Ratio exploration/information. On the other hand, the effect on Percent persuade was significant and of similar magnitude at all levels of clinical experience, as was the effect on Persuade (even if *p* value was borderline significant at moderate experience level). In a similar manner, the intervention effect on Ratio collaboration/information was significant only among the low experience subgroup but was of similar magnitude among all groups.

**Table 3:** Intervention effect stratified by clinical experience.

	Low experience (0–9 years) *N* = 11			Moderate experience (10–17 years) N = 11			Extensive experience (20–37 years) *N* = 10		
Outcome variable	Coef.	BSE	p	Coef.	BSE	p	Coef.	BSE	p
Partnership	1.53	0.51	0.003	0.43	0.71	0.54	0.60	0.38	0.11
Empathy	1.50	0.53	0.005	0.17	0.62	0.78	0.08	0.71	0.91
Seeking collaboration	2.13	0.56	<0.0001	3.39	1.70	0.05	1.06	1.04	0.31
Seeking collaboration by eliciting exploration	0.68	0.49	0.16	1.04	0.90	0.24	-a	-a	-a
Open questions	2.03	0.87	0.02	0.83	0.83	0.32	−1.08	1.05	0.31
Giving neutral information	1.95	2.05	0.34	3.39	3.47	0.33	−0.82	1.90	0.67
Persuade	−2.97	1.30	0.02	−2.30	1.31	0.08	−3.02	1.30	0.02
Persuade with permission	0.74	1.26	0.56	0.91	1.29	0.48	0.58	0.96	0.55
Percent open question	26.24	9.06	0.003	−1.30	10.27	0.91	5.02	18.76	0.79
Percent persuade	−20.45	9.14	0.02	−27.35	12.62	0.03	−21.60	10.42	0.04
Ratio collaboration/information	0.25	0.09	0.006	0.31	0.21	0.14	0.20	0.14	0.14
Ratio exploration/information	0.24	0.10	0.01	0.10	0.14	0.50	0.01	0.11	0.93

*Notes*: Each line shows 3 regression models, i.e. one model for each level of clinical experience. All models were adjusted for profession (nurses vs. physicians) and used 1000 bootstrap replications. Coef: regression coefficient, BSE: bootstrapped standard error.

a The model failed since there were no observation of this skill at this level.

## 4 Discussion and conclusion

### 4.1 Discussion

In this study, we tested the effect of a short e-learning program on practitioners' skills to inform patients. Overall, the intervention group demonstrated significantly better communication behaviors, using less Persuasion and more Seeking Collaboration when providing information to patients compared to the control group, indicating a shift in favor of a more patient-centered approach [31]. Contrary to hypotheses, there were no significant effects on the Partnership and Empathy global ratings, and the behavior counts of Open questions, Giving neutral information and Persuasion with permission. Nevertheless, the intervention's effects varied by clinical experience level, with the intervention showing greater, significant effects in Empathy, Partnership, Seeking Collaboration and use of Open Questions among less experienced practitioners (0–9 years).

Completing the e-learning was effective in reducing practitioner persuasive behaviors and increasing their use of collaborative strategies, such as asking permission before informing, checking understanding or asking for the patient's reaction to the received information, as suggested by our Preparation-Information-Integration model. This is important because these skills can have an impact on patient outcome: research has shown that more persuasive, MI-inconsistent behaviors (higher in the control group) are more likely to be followed by patient resistance [16] [17], and that higher patient resistance probably in turn leads to an increase in persuasive and other negative behaviors among health professionals attempting to promote behavior change [32]. This is predictive of no change [33], [34]. Conversely, less persuasive behavior and more collaborative MI-adherent practitioner strategies most probably result in lower levels of patient resistance [35] and are more likely to be followed by patient change talk, a specific client speech that impacts the likelihood of behavioral change [16], [17], [36].

Overall, the intervention did not significantly impact the use of Open Questions, Giving neutral information and Persuasion with permission. This may reflect the limited statistical power of the study design, given its small pilot sample. It may also indicate that a 1.5-h e-learning program is not sufficient to produce significant changes in such specific communication skills. More experiential training with feedback and coaching has been recommended to consolidate competence [35], [37], [38].

However, the intervention was associated with higher levels of Empathy, Partnership, Seeking Collaboration, and Open Questions among practitioners with less than 10 years of clinical experience. These results suggest that less experienced practitioners may be more amenable to adopting new communication approaches after a 1.5-h e-learning program, possibly because their professional routines are less consolidated, and that practitioners with longer experience may have more difficulties to reassess their established skills and/or implement new techniques and perspectives. It has been suggested that inexperienced practitioners can be more stressed and less open to communication issues than more experienced ones because they are still struggling with acquiring knowledge and technical skills [39], [40]. Our findings, on the other hand, support the integration of a brief e-learning program on information-giving into early stages of professional training to strengthen skill acquisition. This is also in line with the idea that less experienced, thus younger, staff, because of their greater familiarity with technology, are more likely to respond to an online teaching format than older staff [41].

In summary, teaching practitioners to pay attention to the information giving process is possible via a short training program that specifically addresses this skill. In a hospital context, these findings can encourage hospital management to implement this type of training across departments and professions. A similar initiative, albeit with a longer and more intensive communication skill program, was developed in a large hospital in Denmark and yielded positive efficacy and effectiveness results [42]. A short, online course is less time- and resource-consuming, thus potentially easier to implement, and can offer practitioners a model for delivering information in a more collaborative and patient-centered manner.

This pilot study has several limitations. First, by their nature, pilot studies are constrained by limited statistical power, which increases the likelihood that genuine effects remain undetected. Second, because we evaluated the impact of the e-learning in the week following the training, we cannot demonstrate long-term skill retention, which is critical to understanding the sustained impact of a training program. In addition, we relied on voluntary participation, possibly introducing an interest bias. Another limitation lies in the absence of blinding procedures for both outcome assessment and group assignment, as participants were aware of their allocation condition, which may have introduced expectancy bias. Finally, we tested the e-learning on a sample of hospital nurses and physicians only, whereas the program was designed for all staff, clinical and non-clinical, who deliver information to patients. Our results may therefore not be generalized and may not apply to other settings and professions.

Despite these cautions, results of this study are encouraging and are supported by various strengths, including the randomized controlled design of our study, the use of a coding scale based on a valid and reliable coding instrument and the excellent inter-rater reliability. Another strength is the use of a simulated patient (SP), widely accepted as a valuable and effective means of teaching communication skills [43], [44], [45]. We deliberately chose a scenario involving sleep hygiene, a common and plausible concern that ensures realistic role play and consistency across interviews, as often used in simulated patient research [46]. Further research should evaluate long-term effects of skill acquisition, as well as the impact on patient outcomes. It should also evaluate the effect of the e-learning on clinical as well as non-clinical staff.

### 4.2 Innovation

This study contributes to the field by providing preliminary evidence for a strengthened framework for how information delivery can be conceptualized, taught, and integrated within institutional practices. Theoretically, this framework conceptualizes information delivery as a structured, interactive process relevant across a range of clinical and non-clinical contexts. It was practically applied through the development of an e-learning tool designed for all staff who routinely convey information to patients. The e-learning program is designed for broad institutional implementation with minimal logistical constraints or additional resources. The present study offers preliminary support to this framework by verifying hypotheses linking this training to the original conceptual model (Preparation-Information-Integration), even in a small, pilot sample. In addition, by evaluating observable behaviors, the study also advances methodological practice beyond the self-reported measures typically used in the field. Taken together with prior studies showing the relevance of clarifying the information-giving process for providers [9], these contributions position this work as an innovative and original addition to research on e-learning in healthcare and to our understanding of information delivery as a teachable, practicable, and observable communication skill. Institutionally, it opens the door to a broader reflection on communication culture within hospitals, emphasizing that information delivery is not merely a transfer of knowledge but an interactional process central to care quality and to provider-patient relationship.

### 4.3 Conclusion

Overall, the 1.5-h e-learning program showed preliminary support to be a valuable intervention for impacting physicians' and nurses' information-delivery skills, especially among those with less than 10 years clinical experience. Implemented widely, a short online program may represent a time- and cost-effective alternative to in-person training, potentially facilitating access to training and supporting providers in delivering information more effectively to patients. While these findings are encouraging, they should be interpreted in light of the study context, which was limited to clinical staff and a sleep hygiene scenario enacted by a standardized patient. Future studies should examine whether similar effects can be observed across other professional groups, for different types of information, and among real patients.

## CRediT authorship contribution statement

**Cristiana Fortini:** Writing – original draft, Supervision, Resources, Project administration, Methodology, Investigation, Conceptualization. **Nina Rodriguez:** Investigation, Formal analysis. **Joseph Studer:** Software, Resources, Methodology, Data curation, Conceptualization. **Nicolas Bertholet:** Writing – review & editing, Visualization, Methodology, Conceptualization. **Jean-Bernard Daeppen:** Writing – review & editing, Conceptualization. **Jacques Gaume:** Writing – review & editing, Writing – original draft, Visualization, Supervision, Resources, Methodology, Formal analysis, Data curation, Conceptualization.

## Funding

This research did not receive any specific grant from funding agencies in the public, commercial, or not-for-profit sectors.

## Declaration of competing interest

The authors declare that they have no known competing financial interests or personal relationships that could have appeared to influence the work reported in this paper.

## Figures and Tables

**Appendix C:** 

Code		MITI 4.2 code description	Applied modifications	Mean (SD)	ICC
**Global scores (1–5 Likert scales):**					
–	Partnership	Extent to which the practitioner actively shares power in the conversation and conveys an understanding that expertise and wisdom about change reside mostly within the client	–	3.0 (1.1)	0.84
–	Empathy	Extent to which the practitioner understands or makes an effort to grasp the client's perspective and experience and conveys that understanding to the client	–	2.7 (1.2)	0.96
–	Cultivating change talk	Extent to which the practitioner actively encourages the client's own language in favor of the change goal, and confidence for making that change	Not used in analysis (no specific hypothesis)	–	–
–	Softening sustain talk	Extent that the practitioner avoids or minimizes a focus on the reasons against changing or for maintaining the status quo	Not used in analysis (no specific hypothesis)	–	–
**Behavior counts:**					
–	Questions	All questions regardless of the nature of the question	- Open questions (open questions encouraging elaboration) - Closed questions (closed questions inviting yes/no or short answers). Of note, closed questions were not analyzed per se, but coded to allow the computation of Percent open question (see below)	2.6 (1.5) 8.5 (4.7)	0.89 0.96
–	Simple reflections	Simple reflections are those that have no added meaning	Not used in analysis (no specific hypothesis)	–	–
–	Complex reflections	Complex reflections typically add substantial meaning or emphasis to what the client has said	Not used in analysis (no specific hypothesis)	–	–
–	Giving information in a neutral manner	Information of any nature given in neutral language with a neutral tone that does not imply that the client should act on that information	–	6.9 (3.7)	0.87
–	Persuade with permission	Persuade with Permission is assigned when the interviewer attempts to change the client's opinion, attitude, or behavior but does so with permission from the client	–	2.8 (1.8)	0.83
–	Affirmations	Affirmations accentuate something about the client's strengths, efforts, intentions, or worth	Not used in analysis (no specific hypothesis)	–	–
–	Seeking collaboration	Seeking Collaboration includes asking permission to give information or advice, negotiating the agenda of the session, and asking the patient to react to the information	- Seeking collaboration without eliciting exploration (including asking permission to give information or advice, negotiating the agenda of the session, checking integration of the information) - Seeking collaboration by eliciting exploration (inviting the client to reflect on the impact of the information or advice)	2.2 (2.2) 0.4 (0.9)	0.95 1.00
–	Emphasizing autonomy	These are statements that clearly focus on the client's responsibility for decisions, sense of control, or freedom of choice	Not used in analysis (no specific hypothesis)	–	–
–	Persuade	The practitioner makes overt attempts to change the client's opinions, attitudes, or behavior	–	2.8 (3.2)	0.95
–	Confront	This code is used when the practitioner argues with, corrects, shames, blames, criticizes, or warns the client	Not used in analysis (no specific hypothesis)		–
**Summary measures:**					
–	Percent open question	–	Number of Open questions divided by the sum of all questions (closed and open).	26.2 (19.3)	0.76
–	Percent persuade	–	Percent of persuasion when providing information and advice. Computed as the number of Persuade divided by the sum of Persuade, Persuade with permission, and Giving information in a neutral manner.	21.9 (23.7)	0.95
–	Ratio collaboration/information	–	Ratio of seeking collaboration when providing information and advice. Computed as the sum of Seeking collaboration (by and without eliciting exploration), divided by the sum of Persuade, Persuade with permission, and Giving information in a neutral manner.	0.2 (0.2)	0.95
–	Ratio exploration/information	–	Ratio of exploration when providing information and advice. Computed as the sum of Seeking collaboration by eliciting exploration and Open questions, divided by the sum of Persuade, Persuade with permission, and Giving information in a neutral manner.	0.3 (0.2)	0.84

*Notes*: SD: standard deviation; ICC: Intraclass correlation.

## Appendix A Instructions for participants

**You are meeting with Mrs. Julie Weber**, a 46-year-old patient, married with two children aged 11 and 13. You are following up with her in the context of your role within the hospital. You have just finished your usual consultation and still have about fifteen minutes. You ask the patient if there is anything else she would like to discuss, and she uses this time to bring up some difficulties she is having with sleep. Please note that a sleep history has already been taken, and no abnormalities were detected. Today's conversation will focus on lifestyle habits related to sleep.

Your task is to **provide her with information about sleep hygiene**. We will focus on **how** you deliver the information, rather than the **content** of the information itself.

Here are some recommendations regarding sleep hygiene:

• **Maintain consistent bedtimes and wake-up times** – Sleep follows a circadian rhythm (a 24-h biological cycle) that varies from person to person. It's important to respect this rhythm with consistent bedtime and especially wake-up time, even on weekends. A regular wake-up time helps synchronize the sleep-wake cycle.
• **Avoid napping during the day (if you have trouble falling asleep at night)** – If sleep problems already exist, daytime naps can disrupt sleep cycles and should be avoided.
• **Engage in regular physical activity but avoid exercising late in the evening** – Physical activity is beneficial but not recommended late in the evening, as it increases body temperature and stimulates both the body and mind, which can negatively affect sleep. However, an evening walk is perfectly fine.
• **Create a sleep-friendly environment** – Falling asleep is easier under comfortable, quiet, and relatively dark conditions, with a room temperature between 18 °C and 20 °C.
• **Avoid heavy meals, stimulants (coffee, soda, etc.), tobacco, and alcohol before bedtime** – It is recommended to avoid heavy meals late in the evening or just before bed, as well as stimulants like coffee, sodas, *cigarettes*, and alcohol. While some use alcohol as a sleep aid, it leads to poor-quality, unrestorative sleep. A better alternative is herbal tea.
• **Avoid electronic screen light before bedtime** – Using bright screens late at night (especially blue LED lights, unlike red or orange lights) at close range (15–20 cm in the dark) strongly stimulates the retina and disrupts the body's circadian rhythm. It interferes with melatonin production—the sleep hormone—because the body interprets the light as daylight. Exposure to this light affects sleep patterns and impacts neurobehavioral functions (such as increased alertness and brain activity). Effects may vary depending on the distance from the screen, its brightness, and the room's lighting.

## Appendix B Simulated patient role description.

**You are Mrs. Julie Weber**, a 46-year-old woman, married with two children aged 11 and 13. You work one hour away by car from your home. You leave early in the morning and return late in the evening. At the end of your workday, when you have to drive home, you feel tired. You drink coffee to avoid falling asleep at the wheel.

You have a desk-based job, so you are not physically active during work hours. At home, you take care of your two children and your household. Your husband also has long workdays, and you take on most of the family responsibilities. After shopping, looking after the kids, making dinner, etc., it's already late. You're tired, but you feel the need to take a moment for yourself before falling asleep.

About once or twice a week, you go for a run in the evening (after a light meal), covering 8–10 km at a good pace. On those nights, your husband puts the children to bed. After these runs, you go straight to sleep. You tell yourself that a good “physical” fatigue will surely help you fall asleep more easily.

On other nights, you go to bed but spend some time browsing social media to catch up with loved ones or the news; or you watch a series on Netflix to relax. But when it's time to turn off the lights, your eyes are wide open, and you struggle to fall asleep.

You know that too much screen time isn't good, but you're not exactly sure why. Sometimes you tell yourself, “I'll check my phone for 10 minutes, then switch to my book, it's better than screens,” but in the end, you've spent 45 min on social media… You eventually fall asleep very late, and the next morning you have to wake up early. You feel like you're not getting enough sleep. And you know that if you slept more, you'd feel more at ease on the road and probably be more effective at work.

Today, you are seeing the nurse or doctor who is following up with you in the context of their specific role within the hospital. The consultation is shorter than usual, and there are about fifteen minutes left during which you can bring up a topic that concerns you. You want to use this time to talk about your sleep.

**Your opening sentence:** “I know we already talked about this, and that all the tests that were done came back negative, but I'm still having trouble sleeping and feeling tired during the day. On weekends I take a nap to try to recover a bit, but during the week, at work, that's not possible.”
